# A safety study of ultra‐high dose rate FLASH radiotherapy in the treatment of superficial skin tumors: study protocol of a phase I trial (ChiCTR2400080935)

**DOI:** 10.1002/pro6.70010

**Published:** 2025-04-05

**Authors:** Chengliang Yang, Hui Luo, Ma Leijie, Ronghu Mao, Hongchang Lei, Yanping Zhang, Meng Xu, Yiwu Wang, Mingxia Wu, Han Liu, Peng Chen, Hong Ge

**Affiliations:** ^1^ Department of Radiation Oncology The Affiliated Cancer Hospital of Zhengzhou University& Henan Cancer Hospital Zhengzhou China

**Keywords:** Radiation‐associated adverse events, Safety, Superficial skin cancer, ultra‐high dose rate FLASH radiotherapy

## Abstract

**Objective:**

Ultra‐high dose rate FLASH radiotherapy (FLASH‐RT) is emerging as a novel technique to improve the normal tissue tolerance by delivering ultra‐high dose rate radiation several orders of magnitude higher than convention radiotherapy. It has been shown in preclinical studies to cause less injury to surrounding normal tissues during radiation treatment, while still maintaining local tumor control. The purpose of this protocol is to evaluate the safety of fractionated FLASH‐RT in skin cancer.

**Method:**

Patients with superficial skin tumors will be enrolled. The eligible patients will undergo electron FLASH‐RT (24‐40 Gy/3‐5 fractions) to the tumor volume. The primary outcome is to evaluate the safety of FLASH‐RT by collecting the acute (< 90 days) skin toxicity adverse events of radiation according to Common Terminology Criteria for Adverse Events (CTCAE) version 5.0. Secondary objectives include late (> 90 days) skin toxicity after FLASH‐RT according to CTCAE version 5.0 and treatment response.

**Discussion:**

If the results show that delivering FLASH‐RT is safe and feasible for skin tumors, further investigation will be conduct to evaluate efficacy of FLASH‐RT in a phase II trial.

**Trial Registration number:**

ChiCTR2400080935. https://www.chictr.org.cn/showproj.html?proj=220336

## Graphical abstract

FLASH‐RT has been shown to protect normal tissues, the clinical translation of FLASH‐RT is ongoing.

We designed a phase I clinical trial to estimate the safety and healthy tissue spare by using a modified clinical linear accelerator

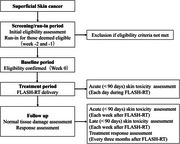

## 1 INTRODUCTION

Skin cancer is one of the most common cancer across the world, squamous cell carcinoma and basal cell carcinoma are the two major types of nonmelanoma skin cancer.^1^ Radiotherapy is an important component for the management of nonmelanoma skin cancer, both in the definitive and palliative setting.^2^

Over the last 30 years, conventional radiotherapy is delivered in fractions to maximize the destruction of malignant cells while minimizing damage to healthy tissues. Technological innovations in radiation oncology led to the development of precision radiotherapy, and the capability of accurately and safely delivering complex‐shaped dose distributions has been improved in conventional radiotherapy.^3^ Unfortunately, the overall clinical response rate of radiotherapy remains poor. Escalation of radiation doses to cancer cells is a promising method to sterilize any tumor and achieve nearly 100% of tumor control. However, the surrounding healthy tissue damage is a key challenge for the delivery of high dose of radiation.^4^

FLASH‐RT is radiation treatment delivered at ultra‐high dose rates (mean dose rate ≥ 40 Gy/s) compared to conventional radiation treatment (usually ≤ 10 Gy/min).^5^ The radiation dose can be delivered almost instantaneously in milliseconds after FLASH‐RT, and this dose rate are believed to cause less damage to normal tissue, also called as FLASH effect.^6^

In recent years, the FLASH effect has been reported in various animal models with different types of irradiations including electron, photon, and proton.^6^, ^7^, ^8^ Although the mechanism of FLASH‐RT in alleviating normal tissue damage is still under investigation, oxygen depletion, peroxyl radical recombination, and immune cell protection are supposed to be essential for the FLASH effect.^5^ This dramatic increase of the differential effect between tumors and normal tissues accelerated its clinical translation. In 2019, Lausanne University Hospital (CHUV) performed electron FLASH‐RT in a patient combined with multi‐resistant CD30+ T‐cell cutaneous lymphoma, there was favorable outcome in this case both on normal skin and the tumor; the results indicated electron FLASH‐RT is feasible and safe.^9^, ^10^ Furthermore, Cincinnati Children's Hospital Medical Center reported the feasibility and safety of proton FLASH‐RT in patients with extremity bone metastases in FAST‐01 clinical trial.^11^ These findings supported the further evaluation of FLASH‐RT in patients with cancer.

The aim of this Phase I study (ChiCTR2400080935) was designed to describe and evaluate the toxicity and efficacy of FLASH‐RT in patients presenting localized superficial skin tumor requiring a radiation treatment.

## 2 METHODS

### 2.1 Objectives

The primary objective of this study is to evaluate the safety and acute radiation‐related toxicity (≤ 90 days) in patients with localized superficial skin tumor after FLASH‐RT using National Cancer Institute (NCI) Common Terminology Criteria for Adverse Events (CTCAE v5.0).^12^

The secondary objectives are as follows: 1. To evaluate late toxicity (> 90 days) of FLASH‐RT in patients with localized superficial skin tumor from time of radiation. 2. Estimate treatment efficacy of FLASH‐RT with the Response Evaluation Criteria in Solid Tumors (RECIST) criteria (v1.1) after radiotherapy.^13^

### 2.2 Study design

**FIGURE 1:**
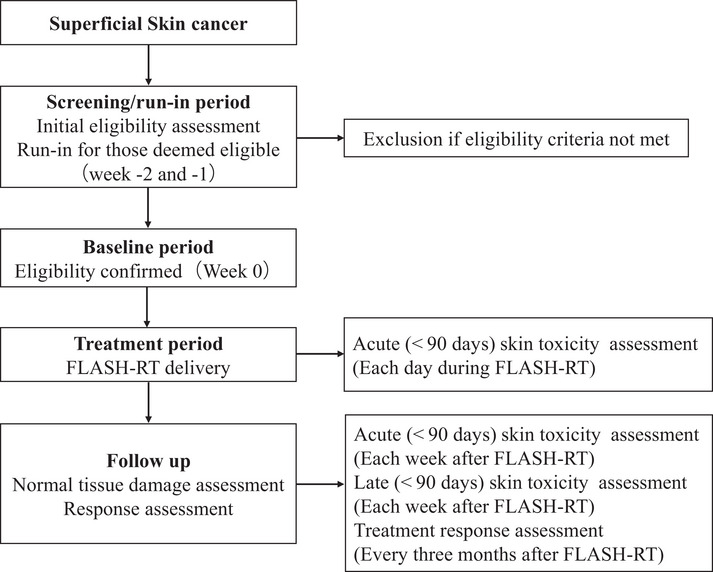
Study protocol schema. FLASH‐RT, Ultra‐high dose rate FLASH radiotherapy.

This is a single institution, prospective, single arm safety study to evaluate the feasibility and safety of electron FLASH‐RT in patients with localized superficial skin tumor. Patients with localized superficial skin tumor will be considered for enrolling in this phase I study. All the eligible patients will be discussed at multi‐disciplinary meeting. Patients will be formally recruited and managed by members of the FLASH‐RT team. The treatment protocol is shown in Figure 1.

### 2.3 Interventions (FLASH‐RT)

The radiation plan for eligible patients are as following: For T1 (small) lesions: 3×8 Gy fractionated dose FLASH‐RT; For T2 (large) lesions: 5×8 Gy fractionated dose FLASH‐RT. FLASH‐RT will be delivered to all patients using 9 MeV electrons. The parameters were as following: source skin distance, 100 cm; instantaneous dose rate, 82500 Gy/s; average dose rate, 120 Gy/s; pulse width, 4.0 µs; frequency, 360 Hz; pulse number, 360. Detail of the linear accelerator used for FLASH‐RT were reported previously (supplementary Figure 1).^14^ All the parameters were measured and certificated by China Institute of Atomic Energy. Dosimetric checks were conducted before, after and during FLASH‐RT. The dose distribution of FLASH‐RT will be measured by both alanine pelets and Gafchromic HD‐V2 Film.

### 2.4 Rational for radiation dose

The total radiation doses delivered for tumors were: 24 Gy in 3 fractions (biologically effective dose (BED_10_) = 43.2 Gy) for T1 disease, and 40 Gy in 5 fractions (BED_10_ = 72.0 Gy) for T2 disease. This dose was applied because BED_10_ between 40 and 72 has been associated with excellent overall cure rates for superficial skin tumor.^2^

### 2.5 Follow‐up

Radiation‐induced adverse events will be assessed according to NCI‐CTCAE (v5.0).^12^ Acute toxicity will be monitored and recorded on a weekly basis during the first 3 months. Late toxicity will be evaluated and recorded thereafter. Treatment response will be assessed by using the RECIST criteria (v1.1).^13^ Patient follow‐up is typically scheduled in clinic every 3 months for the first 2 years post‐FLASH‐RT and every 6 months thereafter by performing physical examination, computed tomography imaging, and routine laboratory test.

### 2.6 Inclusion criteria

Patients must fulfill all the following criteria to be eligible for inclusion in this study:

(1) Age: 18–75 years old. (2) Patients with histologically proven cutaneous squamous cell carcinoma or basal cell carcinoma. (3) Without distant metastasis. (4) Without any other medical condition or laboratory value that would, at the discretion of the investigator, preclude the patient from participation in this clinical investigation. (5) Without any serious underlying medical condition that could interfere with study treatment and potential adverse events. (6) The Eastern Cooperative Oncology Group (ECOG) score: 0–2. (7) Patients requiring radiotherapy treatment according to the dermato‐oncology tumor board: standard treatment is ineffective, patients who are either medically inoperable or refuse to surgical resection, and/or anatomical locations where surgery can compromise function or cosmesis. (8) The disease progression affects patients’ quality of life. (9) T1‐T2 N0 lesions with a small (T1; lesion ≤ 2cm in diameter) or large (T2; 2cm < lesion ≤ 4 cm) volume (the Union for International Cancer Control (UICC) Classification of Malignant Tumours, 8th Edition); the size of the treated lesion should be ≤ 3.5 cm thick (caliper‐based measurement). (10) Clinically acceptable treatment plan with electron FLASH‐RT. (11) Treatment associated side effects are limited and controllable. (12) Life expectancy ≥ 3 months (in the judgement of the investigator). (13) A FLASH‐RT team consisting of 4 radiation oncologists, 3 radiation physicists, and 1 dermatologist should be established prior to radiotherapy. (14) No antibiotics, hormones, immunosuppressive drugs, probiotics, etc. were used within 1 month prior to treatment. (15) Patients who can comply with the protocol. (16) Sign the phase I study informed consent form.

### 2.7 Exclusion criteria

Patients who meet any of the following criteria will be ineligible for this study: (1) Patients who are pregnant or nursing. (2) Patients with known contraindications to radiation. (3) Concomitant use of systemic oncological treatment for a cancer other than the skin cancer. (4) Concomitant auto‐immune disease with skin lesions. (5) Patients who will receive cytotoxic chemotherapy within 1 week prior to or 1 week following their planned radiation treatment. (6) Previous radiotherapy in the treated area. (7) Concomitant use of radio‐sensitizer drug. (8) Patients who are unable to comply with study requirements or follow‐up schedule. (9) Patients who combined with cognitive disorders not compatible with the signature of informed consent or other impairment that may compromise compliance with the requirements of the study. (10) Patients with symptomatic pneumonitis at the time of screening, or a history of symptomatic radiation pneumonitis. (11) Patients enrolled in any other clinical studies the investigator believes to conflict with this clinical investigation.

### 2.8 Statistical analysis and sample size

Descriptive statistics will be utilized to record baseline and clinical pathological parameters. The sample size of our study was 12 patients, and data analysis will be conduct once the study has been completed.

### 2.9 Safety stopping rules

FLASH‐RT will be delayed until toxicity improves to less than grade 2 in case any of the following adverse events: White blood cell count < 1.5 × 10^9^ cells/L; Absolute neutrophil count < 1.0 × 10^9^ cells/L; Platelets < 40 × 10^9^ cells/L; ≥ grade 3 non‐hematological toxicity.

If skin infection is observed in the tumor area with fever over 38.5°C, FLASH‐RT will be delayed until complete recovery. And FLASH‐RT will be terminated if the patient does not recover within 2 weeks.

### 2.10 Ethics and dissemination

This study was conducted in accordance with the ethical standards of the World Medical Association Declaration of Helsinki. The study was approved by the Medical Ethics Committee of Henan Cancer Hospital & the Affiliated Cancer Hospital of Zhengzhou University (2023‐KY‐0148‐003). The written informed consent was approved by the review board of our institution. All patients or an authorized representative, will be informed of the nature of the study and will provide the written informed consent. The risks, benefits, and details of each intervention will be discussed by the radiation oncologists with all patients enrolled on this protocol. This study has been registered at Chinese Clinical Trial Registry.gov (ChiCTR2400080935). The results of this trial will be submitted to a peer‐reviewed journal for publication. The abstract will be presented on international conferences.

## 3 DISCUSSION

Radiotherapy could be regarded as the primary treatment in patients of superficial skin tumor that are not eligible for surgery due to advanced tumor stage, complications, location of lesions, and comorbidities.^2^ The most common treated area includes forehead, nose, cheeks, lips, chin, and scalp.^15^ Tumor location can have significant cosmetic consequences, and radiation‐related adverse effects is one of the risk factors for obtaining excellent cosmetic results.^2^

The rapid progress in technology, physics, and computer sciences promote the development of radiation oncology. FLASH‐RT has been recognized as one of the most promising breakthrough technologies that will change conventional radiotherapy. FLASH‐RT can be delivered in extremely short time (usually in milliseconds) and substantially can alleviate radiation‐related adverse effects, thus, widen the therapeutic window of RT.^5^ These advantages made FLASH‐RT as an attractive strategy for localized SUPERFICIAL SKIN TUMOR.

Indeed, the Group from Lausanne University Hospital used a single dose of 15 Gy electron FLASH‐RT (average dose rate 166 Gy/s, delivered in less than 100 milliseconds) in the patient combined with cutaneous lymphoma, a maximal of grade 1 adverse effects (epithelitis and edema) was observed between day 10 and 44; complete tumor response was achieved 36 days after FLASH‐RT and without recurrence thereafter.^9^ At present, there are two ongoing clinical trials performed by the group using electron FLASH‐RT for skin cancer. One is a single center phase I, first‐in‐human, dose escalation study of FLASH therapy (NCT04986696) in patients with skin melanoma metastases that progress locally despite systemic treatments (7 dose levels: 22 Gy; 24 Gy; 26 Gy; 28 Gy, 30 Gy, 32 Gy and 34 Gy). The other is a Phase II study of FLASH‐RT (NCT05724875) versus conventional RT in patients with localized cutaneous squamous cell carcinoma or basal cell carcinoma (a single dose of 22 Gy for T1 lesions and 5×6 Gy fractionated dose for T2 lesions).

Besides, the group from Cincinnati Children's Proton Therapy Center performed proton FLASH‐RT (NCT04592887) for the palliative treatment of painful bone metastases, a total of 10 patients was included and a single dose of 8 Gy was delivered.^11^ Acute treatment‐related toxicities of FLASH‐RT including grade 1 edema, erythema, fatigue, pruritus, skin hyperpigmentation and grade 2 extremity pain. Long term toxicity of grade 1 skin discoloration was only observed in 1 patient. Overall, the adverse events of proton FLASH‐RT were mild and consistent with conventional RT. This group is conducting another clinical trial to assess toxicities of FLASH‐RT (NCT05524064) and pain relief in patients with painful thoracic bone metastases.

In conclusion, radiation oncology is on the way to the era of FLASH‐RT. The present protocol will evaluate the toxicity of electron FLASH‐RT for the treatment of localized superficial skin tumor. If the results show that FLASH‐RT is safe and feasible, it warrants further investigation in the future.

## CONFLICT OF INTEREST STATMENT

The authors declare that they have no known competing financial interests or personal relationships that could have appeared to influence the work reported in this paper.

## ETHICAL STATEMENT

This study was conducted in accordance with the ethical standards of the World Medical Association Declaration of Helsinki. The study was approved by the Medical Ethics Committee of Henan Cancer Hospital & the Affiliated Cancer Hospital of Zhengzhou University (2023‐KY‐0148‐003). The written informed consent was approved by the review board of our institution. All patients or an authorized representative, will be informed of the nature of the study and will provide the written informed consent.

## Supporting information



Supporting information

## Data Availability

Datasets and other files generated, analyzed, or processed in this study are available upon request from the corresponding author.
