# Supplementary material for: A safety study of ultra‐high dose rate FLASH radiotherapy in the treatment of superficial skin tumors: study protocol of a phase I trial (ChiCTR2400080935)
Source: Precis Radiat Oncol. 2025 Apr 5;9(2):72–6. doi: 10.1002/pro6.70010 (PMC12559900; doi:10.1002/pro6.70010)
Supplement: Supplementary file 1 — Supporting information [file PRO6-9-72-s001.docx]

**Fig. 1** The modification of Varian 23CX clinical accelerator to establish a FLASH-RT platform. (A) The modification of Varian 23CX clinical accelerator. (B) The isodose line distribution of 9 MeV electron after modification. (CONV-RT, conventional radiotherapy; FLASH-RT, ultra-high dose rate FLASH radiotherapy)


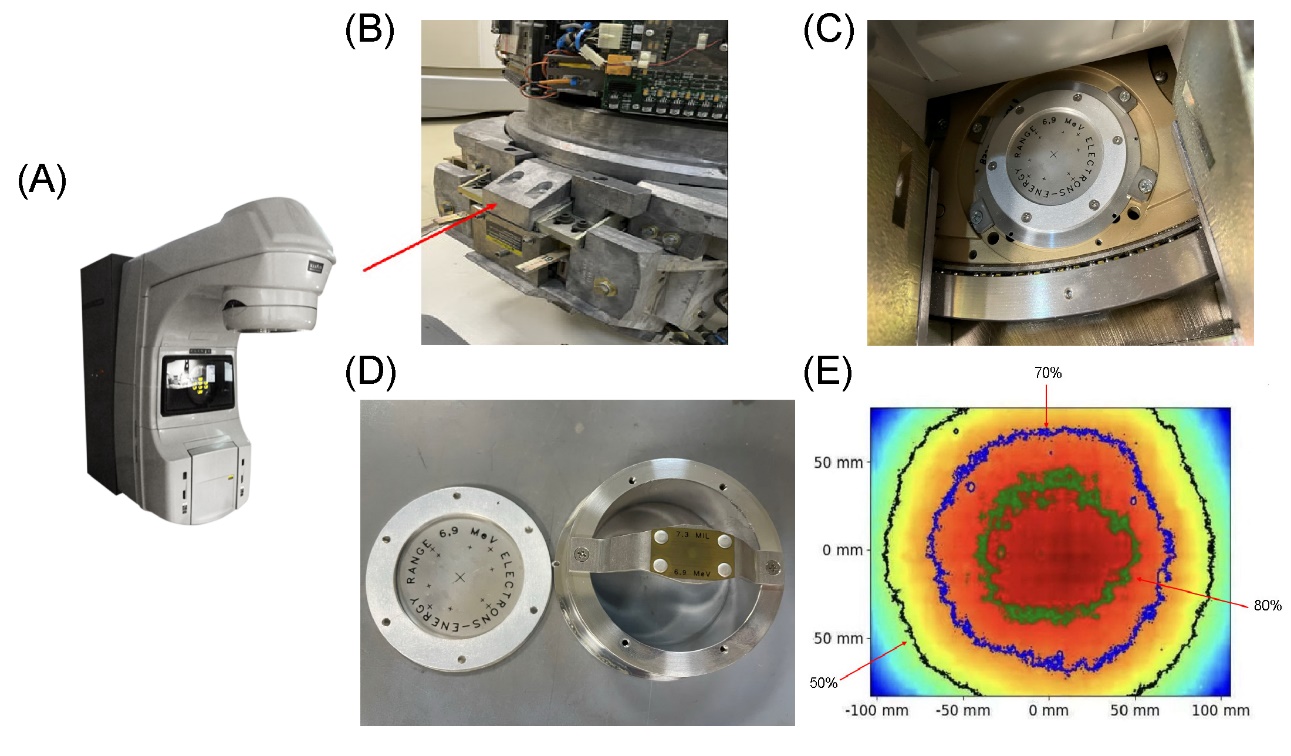


**REFERENCE**

Ma LJ, Mao RH, Lei HC, Ge H, Chen P, Liu H, Li B, Luo H. Transform routine medical accelerators to achieve Flash-RT and physical performance measurements. Chin J Radi Oncol. 2022;31(11):1022-1027.
